# From ‘Implications’ to ‘Dimensions’: Science, Medicine and Ethics in Society

**DOI:** 10.1007/s10728-012-0219-y

**Published:** 2012-09-05

**Authors:** Martyn D. Pickersgill

**Affiliations:** Centre for Population Health Sciences, Medical School, University of Edinburgh, Teviot Place, Edinburgh, EH8 9AG Scotland, UK

**Keywords:** Biomedical technology, Biomedicine, Empirical bioethics, Innovation, Science and technology studies, Sociology of bioethics

## Abstract

Much bioethical scholarship is concerned with the social, legal and philosophical implications of new and emerging science and medicine, as well as with the processes of research that under-gird these innovations. Science and technology studies (STS), and the related and interpenetrating disciplines of anthropology and sociology, have also explored what novel technoscience might imply for society, and how the social is constitutive of scientific knowledge and technological artefacts. More recently, social scientists have interrogated the emergence of ethical issues: they have documented how particular matters come to be regarded as in some way to do with ‘ethics’, and how this in turn enjoins particular types of social action. In this paper, I will discuss some of this and other STS (and STS-inflected) literature and reflect on how it might complement more ‘traditional’ modes of bioethical enquiry. I argue that STS might (1) cast new light on current bioethical issues, (2) direct the gaze of bioethicists towards matters that may previously have escaped their attention, and (3) indicate the import not only of the ethical implications of biomedical innovation, but also how these innovative and other processes feature ethics as a *dimension* of everyday laboratory and clinical work. In sum, engagements between STS and bioethics are increasingly important in order to understand and manage the complex dynamics between science, medicine and ethics in society.

## Introduction

Bioethics has long been a multidisciplinary enterprise.^1^ Increasingly, social science methods, approaches and perspectives are deemed to have relevance for or are even integrated into the bioethical enterprise. This is reflected in and further stimulated by recent moves towards ‘empirical bioethics’ [26, 27, 40]. Science and technology studies (STS) is one such tradition that is articulating with bioethics, though sometimes fractiously [9, 24]. In this paper I discuss what benefits bioethics might afford from (more) engagement with STS, whilst also highlighting some of the challenges to such rapprochements.

The contributions that STS has made, and will continue to make, to our understandings of science and medicine are diverse. This is as a consequence of the range of conceptual approaches drawn upon within investigations, and the diverse methods that have been employed (including participant observation, focus groups, interviews, and documentary analysis which commonly take scientific articles and sites of knowledge production and governance as their empirical object). Social science research that either engages with or is directly situated within the STS literature has, in particular, cast new light on a range of issues associated with bioethical questions and concerns. This includes even the occasionally contested field of the sociology of bioethics (which might be regarded as an area of scholarship nested within the broader STS ‘canon’).

Here, I map out some—but by no means all—of the contributions STS might make to bioethics. Broadly, I characterise these as the capacity of STS to provide us with an augmented understanding of the social worlds of those who engage in practices we deem un/ethical, and to identify problems which might be overlooked by bioethicists. Furthermore, empirical STS research challenges the focus of bioethical scholarship concerned solely with the ‘implications’ of biomedicine (i.e. what effects innovation might and should have on science, medicine and society). It does this through highlighting the diverse ‘regimes of normativity’ [54] within which actors are embedded, and the degree to which moral and ethical decision-making and action is a constitutive dimension of work and everyday life.

## What is STS?

What, exactly, comprises the definitive quality of STS has long been—and continues to be—a key question that vexes its practitioners and creates confusion in many who encounter the field for the first time. In some ways, however, the ambiguous identity of STS might, in fact, induce the comfort of the familiar in bioethicists, who have long sought to define their purview and wrestle with what, if anything, separates their enterprise from moral philosophy or law. A key text within STS that is central to the self-identity of the field is the volume, The Handbook of Science and Technology Studies; here, STS is defined as “an interdisciplinary field that is creating an integrative understanding of the origins, dynamics, and consequences of science and technology” [18: 1]. Broadly, it is concerned with the creation, standardisation, circulation, governance, implementation and claims to expertise regarding knowledge and technology.

In effect, STS is at once a specific disciplinary field and an interdisciplinary milieu. Incorporating sociology and anthropology, as well as history, linguistics, philosophy and political science (and perhaps bioethics), STS scholars employ methods and concepts from all of these traditions. What is perhaps different in STS is its predominant emphasis on the use of case studies to produce theory, rather than testing theory using cases; however, this is perhaps a difference in perspective and approach rather than an indication of sharp boundaries between STS and (for instance) anthropology and sociology [38]. Furthermore, STS and other social scientists draw on similar theorists (e.g. Mary Douglas, Michel Foucault, Harold Garfinkel) and interweave insights that ‘belong’ to diverse traditions [38]. At the same time, however, STS has its own journals, professional associations, and specific conceptual vocabulary. In the latter case, actor-network theory (ANT) is an especially noteworthy example; developed by Michel Callon, Bruno Latour and John Law, ANT has travelled from STS to be incorporated in various other traditions, including socio-legal studies, history, and health services research.^2^

Several other terms and phrases used in STS that a range of practitioners from other disciplines may be familiar with also have specific meanings when deployed within the field’s specialist journals. A key example here is the idiom of ‘co-production’, which STS scholars use to refer to as

the proposition that the ways in which we know and represent the world (both nature and society) are inseparable from the ways in which we chose to live in it. Knowledge and its material embodiments are at once products of social work and constitutive of forms of social life; society cannot function without knowledge anymore than knowledge can exist without appropriate social supports. Scientific knowledge, in particular, is not a transcendent mirror of reality. It both embeds and is embedded in social practices, identities, norms, conventions, discourses, instruments and institutions – in short, in all the building blocks of what we call the *social*. The same can be said even more forcefully of technology [29: 2–3].

We can likewise say the same of medicine—itself a complex assemblage of knowledge, technology and sociality. Within STS such matrices are objects of study; in particular, STS scholars are attentive to the ways in which citizens are enrolled into bioscience in terms of how individuals and social groups direct research and shape innovation, as well as to how public voices and concerns may be silenced (from the level of the specific research encounter, to scientific governance). The very meaning of ‘the social’, however, is contested within STS; in comparison to much but by no means all of more ‘conventional’ social science, sociality is understood as being constituted through and enabled by materiality (such as interactions with technologies and other artefacts). In sum, then, the boundaries between STS and disciplines such as (medical) anthropology and sociology are not always clear (hence, literature that is not solely ‘pure’ STS—if indeed there is or should be such a thing—will be considered in the analysis presented here), but in general STS has a greater emphasis on science and technology as specific empirical ‘objects’ than other areas of social science. Moreover, STS has a heightened sensitivity to the ways in which these produce the social domains other disciplinary scholars describe and seek to explain.

### The Sociology of Bioethics

One particular focus of STS literature has been the sociology of bioethics; that is, the study of the social life of bioethical problems, the role of knowledge and technology in structuring and defining these, the political economy of the solutions reached, and the methods by which they are achieved. Particular foci of work in this vein are explorations of the place, role and impact of public bioethics in policy and biomedicine [32, 48, 61, 62], and examinations of the mutual reinforcement—and perhaps co-production—of social and epistemic innovation in regards to controversial and/or promissory technoscience [22, 23]. Here, the reciprocal formation of neuroscience and neuroethics is a salient case in point [6, 7].

In part, we might see the rise of the sociology of bioethics as being linked to the expansion and growing prestige of the bioethical enterprise itself. Moreover, the attention of STS researchers to bioethics can, to an extent, be viewed as symptomatic of a wider debate within the social sciences about ethics regulation (see [10, 20]). STS has shown itself to be finely attuned to identifying and interrogating forms of technoscientific praxis that have deep traction within society (molecular biology and climate science being important examples); accordingly, interest in bioethics should not be surprising in light of the increasing institutional power of bioethical questions, actors and networks.

It is likewise unsurprising that some bioethicists might take STS in general and the sociology of bioethics in particular to be critical of their enterprise. As the so-called ‘Science Wars’ of the 1990 s might remind us [70], influential individuals do not always take kindly to having the ‘black box’ [55] of their work unpacked and its contents inspected. Furthermore, it is clear that STS scholarship in this area does often contain critique; for instance, bioethics has been read as being ‘too close’ to science, compromising its objectivity, and providing legitimacy for controversial scientific endeavours.^3^ More generally, the emphasis of STS on expertise might also be deemed problematic to bioethics; research orientated towards deconstructing not only the knowledge claims of bioethicists but also who is legitimately entitled to expound them leads to the ‘problem’ of potentially undermining the status of the bioethicist as an expert who occupies a privileged role in the governance of biomedicine.^4^

However, such critique does not have to be read as negative. Rather, STS, as a field, might be regarded as being a ‘critical friend’ to and interlocutor with bioethics. In what follows, I outline some of the diverse contributions that STS might make to the bioethical enterprise.

## What Might STS Contribute to Bioethics?

The literatures and approaches from STS and broader social science that might afford benefit to bioethicists is diverse; however, of particular note is recent scholarship on the place, role and impact of biomedical technologies in medicine and wider society, and the production and consumption of drugs.

### Casting New Light

In regards to the former, methods of visualising the interior of the body—such as magnetic resonance imaging (MRI)–have been shown to be ascribed an authority which encourages their use even when they are costly and do not demonstrate clear therapeutic benefit [31]. In turn, articulations of benefit itself come to be a function of the nature of disease as mediated and understood through biomedical technique [46]. Such findings prompt further reflection and debate over health care rationing and resource allocation, and access to biomedical innovation. As bioethicists Martin and Singer [45] point out, priority setting in medicine must include some form of descriptive analysis, and empirical STS research can help to create a new vantage point from which the use of resources can be viewed.

Alongside the shifts in the ontology of pathology that Mol [46] and others have shown comes with the introduction of new health technologies, transformations in the meanings of care can also occur. As pointed out by Dick Willems [68], a medical ethicist with an STS-orientation, the introduction of novel technologies helps to constitute new kinds of caring practice. Likewise, we also see fresh challenges to simplistic understandings of patients’ ‘choices’ in regards to their use of biomedical tests and tools [37]. This raises questions about how to mandate and monitor ‘good’ care. In part, this is because what precisely care is can be mutable and highly context-specific; furthermore, the ways in which ‘good’ care may entail practices of coercion can be complex. Such matters are important for health professionals and ethicists to continue to explore, not least as a consequence of how highly regulated standards of care currently are in many countries. The value STS affords bioethics here is its empirical, case-study approach, which enables the careful evidencing of how agency and autonomy, technology and standardisation, and caring practices all shape each other.

The extent to which health technologies can escape the rubric of biomedicine and become enrolled within wider cultural regimes (such as the criminal justice system) also bears further attention. As Melissa Littlefield [43] has documented, MRI has left the hospitals and laboratories where it is more commonly located, and can now be found in the courts. These translations rely not solely on particular perceptions of the technology, but also ideas and assumptions about society and socio-legal processes that may be questionable—yet which nevertheless can become reified through performance [51]. Such findings add empirical weight to theoretical but practically-orientated bioethical scholarship concerned with the governance of science, and the diffusion and consequences of innovation. Detailed STS investigations of technology transfer (in its broadest sense) are likewise relevant to a variety of matters currently vexing bioethicists, including human enhancement. Historical and contemporary studies of how and why artefacts travel might usefully contribute to more grounded analyses of the promises and perils of technologies that can or could enhance the body.

### Expanding the Bioethical Gaze

The considerations of agency and autonomy that are so central to ethical appraisals of biomedical technologies are likewise key issues in relation to psychopharmaceuticals [15, 64]. Yet, wider changes in pharmaceutical consumption also direct our attention to less frequently regarded ethical issues around the innovation, testing and circulation of drugs. Social scientists have increasingly focused on such matters, and their scholarship could have import for bioethics. For instance, Petryna’s [50] work on the outsourcing of clinical trials to middle and low income countries has revealed a range of problematic developments, including biased trial designs that ensure drugs look safer and more efficacious, and proceduralism in ethical review and administration that “can hide contextual uncertainties” [50: 187]. However, anthropological and sociological studies of biomedicine highlight that such problems are not solely salient in contexts beyond ‘the West’. Rather, as Abadie [1] starkly illustrates, participation in trials in the US can likewise involve what Singh [65] might call ‘cryptic coercion’—as well as more overt forms. Practices of coercion and the strategies of resistance that these impel may impact in important ways on the knowledge trials seek to produce, with a number of ethically significant consequences.

More generally, in “making doctors familiar with new medicines and fuelling patient demand clinical trials also become powerful marketing tools and can significantly alter local and public health care priorities” [50: 198]. Indeed, as Lakoff [36] has evidenced, trials can contribute to the spread of not solely drugs but the diagnostic categories that they purport to treat (e.g. bipolar disorder). Psychopharmaceuticals themselves are circulating globally, and being positioned not just as remedies for previously unrecognised psychic ailments, but also as tools to fix economic concerns such as ‘presenteeism’ [30]. As Stefan Ecks [12] vividly shows, drugs like antidepressants have *sociotopic* as well as *psychotropic* effects: their use reshapes the spaces within which individuals deemed pathological are allowed to inhabit or enabled to use. Understandings of personhood have also been argued to articulate with drugs and biomedical technologies in diverse ways. For instance, visualisation technologies like positron emission tomography (PET) can support new, explicitly brain-based notions of subjective distress that have had evident effects on activism and public health campaigns [11]. Within the clinic, neurological explanations for opaque conditions can sometimes have traction as a framework through which to deal with the uncertainties associated with them [52]. Technologies and treatments, then, impact on the politics and lived experience of health and illness in important ways that perhaps go beyond traditional concerns in research and healthcare ethics (e.g. beginning and end of life issues, confidentiality, consent and experimentation, liability, resource allocation).

The attention of STS to lived experience may also widen the bioethical gaze in regards to public engagement. Activities in this vein are often used as fora within which to educate non-scientists about biomedical developments whilst also promoting wider discussion of their social and ethical aspects. Yet, often an expert-lay divide is perpetuated which closes down opportunities for more reflexive debate [35]. Accordingly, the fresh perspectives that might be gleaned from public participants and which might have salience to bioethicists are ‘framed out’. In so doing, opportunities for more democratic forms of bioethical deliberation are also restricted [53]. As STS scholars have shown, publics can be both knowledgeable about biomedicine and willing to engage in sustained debate and analysis about issues that bioethicists are grappling with [34, 56]; limiting participation is thus unfortunate not only for democractic reasons, but also because potentially ‘useful’ contributions from those outside the academiy remain unheard. However, some investigators working within bioethics are drawing on STS research and explicitly seeking to enrol wider publics into ethical analysis (e.g. [41, 63]). Such work has the potential to enrich both STS studies of expertise and deliberation, and bioethical frameworks for thinking about the impacts and acceptable limits of biomedical innovation.

### From Implications to Dimensions

STS, then, is useful for bioethicists to engage with on account of the fresh light it casts on the implications of new biomedical techniques and practices, but also as a consequence of the novel and under-examined issues it directs the bioethical gaze towards. However, from a co-productionist perspective, STS and other work in the social sciences also illuminates that though ethical reasoning is most evidently located in discourses on the ‘implications’ of biomedicine, it is also a constitutive dimension of scientific and medical knowledge and practice. As anthropologist Paul Brodwin [5] has demonstrated, for instance, professional ethics and moral discourse intertwine in US psychiatry: sedimenting within clinical work, transforming practice, and being reshaped in the process. Recognising this “essential entanglement of the moral and the factual” [19: 471] is a necessary step to take in order to grapple with bioethical questions that have long been a concern to many in the field, including how scientists and clinicians “actually solve ethical problems and make ethical decisions” [4: 96].

Social scientists have produced a range of works that speak directly to such problematics. For example, Hooeyer [25] has shown how moral qualms around trade in human body parts are managed through systems of ‘compensation’ which ascribe value to biomaterials without the formation of ‘markets’, and Frith et al. [17] have underscored the routine engagement with ethical issues that constitutes clinical practice within the infertility clinic. Indeed, ethical issues may play a key role in the implementation of new technologies within the clinic [21]. In turn, Williams et al. [69] note how ethical concerns or imperatives (e.g. enhancing patient choice) can be compromised in the face of wider changes to healthcare systems that professionals feel powerless to challenge.

We can see likewise see that research trajectories and designs are powerfully impacted not only by formal governance and legislation, but also by the everyday ethics of researchers—as well as by study coordinators and managers who may attempt to use “informal ethical practices” in attempt to “reinsert care into research” [16: 689]. The significance of everyday ethics has been documented, for instance, in studies of controversial areas of investigations such as stem cell science. Within this field, the collection of ‘spare embryos’ is central to research; yet, the construal of an embryo as ‘spare’ must be achieved through careful ethical argumentation and deliberation which is itself experimental [14, 66]. Here, as elsewhere, boundaries between un/ethical forms of investigations are discursively constructed which at one “define and defend the work of scientists involved in ethically sensitive research” [67: 745].

Considering the centrality of ethical behaviour to processes of scientific knowledge production and application also reminds us of the import of ethics for helping to consolidate and drive forward particular kinds of biomedical paradigms (as discussed in the previous section). From this perspective, we can see that ‘ethics’ does not just come after the ‘facts’ of science; rather, it is essential to the forging of these. This has long been a concern of STS scholars, who have shown extensively how scientists have views on the impact of their research on wider society but nevertheless seek to demarcate these from their professional work [13, 33, 49, 54]. Some bioethicists have likewise been attentive to these issues; as Molewijk et al. [47: 87] put it, “science is inherently interwoven with normative issues”.

Accordingly, the function of STS within a bioethics context is not solely to underscore the diverse forms of ‘implications’ that shifts in health research and care both potentiate and activate within society, but it is also to show when, where and how ethics acts a ‘dimension’ of biomedicine. Ethical questions, ethical discourse, and ethical regulation all form a ‘regime of normativity’ [54] within which scientists and health professionals conduct their work, and which shapes (and is shaped by) this.

## Discussion

In this article I have introduced some of the central concerns of STS and related scholarship, and discussed the recent focus of this on bioethics itself. Arguing that the issues STS has raised in regards to the social life of bioethics might be more usefully interpreted not as confrontations but as critical engagements, I then went on to describe some of the ways through which bioethical scholarship might afford benefit from further encounters with a range of STS (and STS-inflected) research. In particular, work on health technologies and pharmaceuticals may cast new light on matters of import to bioethicists, as well as potentially drawing attention to other practices and debates that have perhaps thus far escaped the bioethical gaze. Finally, I have aimed to show that STS and related work, especially medical anthropology and sociology, reveals the extent to which a range of actions (including knowledge production) are structured by ‘ethical’ concerns and produce effects that in turn might raise new ethical questions. ‘Ethics’ is thus a ‘dimension’ of science, medicine and everyday life rather than something that gains salience only after facts are made. Considering the ethical dimensions of science and medicine might bring to light new issues for bioethics to address, whilst potentially also problematizing existing solutions. Bioethical analysis may thus be further enhanced through rapprochement with STS, including through the increasingly interdisciplinary enterprise of ‘empirical bioethics’.^5^ In this vein, several scholars from fields such as bioethics, law, philosophy, sociology and STS have begun to forge networks and relationships that have led to a variety of cross-disciplinary research projects which have yielded findings of relevance to each of the traditions represented.

This latter point reminds us that though this article is primarily concerned with how STS (and work resonant with STS) might inform or contribute to bioethics, it is also necessary to consider the value of the latter discipline to the former. Of most obvious salience here is the essential concern of bioethicists with normativity. Such an emphasis enjoins STS scholars to confront the normative assumptions underpinning their own work; although social scientists are extremely reflexive about such matters, bioethics may potentially contribute to the development of new frameworks through which normativity can be interrogated, articulated and managed. More generally, bioethics could make a contribution to the mapping of new empirical terrain. This might, in part, be through illuminating features within the landscape of medicine and science that STS scholars may have failed to attend to or of which they were hitherto unaware. It will also be as a consequence of the new kinds of questions that collaboration compels investigators to ask; much as conceptual development in the social sciences has been stimulated through close associations with scientists and health professionals, so too might collaborative relationships with bioethics animate innovation in STS.

Nevertheless, challenges remain. Both STS and bioethics are highly diverse fields which lack coherent and uncontested disciplinary identities; this can make mutual understanding difficult, not least because scholars from and within each tradition may approach the same problem quite differently in terms of methods, conceptual underpinnings, and normative agenda. Ironically, in cases where the same or a similar ‘solution’ is reached, the different routes to it that were taken could lead to professional boundary-work and institutional distance rather than further collaboration. This is especially significant at a time when academics are increasingly encouraged to compete for scarce resources and demonstrate the ‘impact’ of their work (for instance, through involvement with regulatory and advisory bodies). Furthermore, the sociology of bioethics is likely to continue to be perceived as an ‘attack’ by some bioethicists (and, indeed, perhaps even intended as such by some STS analysts). Accordingly, the apparent differences between bioethics and STS will need to bear careful and honest scrutiny; in so doing, the disciplines may be found to be less dissimilar than at first appears. For instance, STS indictments of bioethics that it is ‘too close’ to science recall some internal critiques, including those from feminist bioethicists who have sought especially deep critical engagement with biomedical institutions and practices [44, 59, 60] and bioethical scholarship itself [42, 57, 58]. Indeed, STS critiques of bioethics being ‘too close’ to science are, in a sense, normative assertions about the ways that bioethics should (not) be carried out, and thus ultimately claims about how biomedicine should be governed. Is this, we might ask, just another way of doing bioethics? For some, these and related questions pertaining to the convergences and divergences of bioethics and STS will be irrelevant or mundane—but to others they will be anathema. It is precisely because of this that they will need to be articulated and explored, in order that the opportunities and disincentives to collaboration between STS and bioethics are appropriately engaged with and interrogated, and the potential benefits to scholarship realised.

## Footnotes

1 Throughout this paper, I use the terms ‘bioethics’ and ‘medical ethics’ as roughly synonymous, though understanding the latter as pointing to specifically medical contexts rather than biomedicine and the health professions more broadly.

2 For an overview of ANT, and some of the associated debates, see Law and Hassard [39].

3 Although we know from STS itself that objectivity is a social and culturally embedded construct and achievement rather than something that pre-exists attempts to practice it [8].

4 Of course, bioethicists are no unreflecitive in regards to their own claims to expertise; see, for instance, Archard [2] and Ives and Dunn [28].

5 It should not be forgotten that the rise of empirical bioethics itself presents an interesting case for STS to explore. For a related point, see Ashcroft [3].
